# Zinc oxide nanoparticles induces cell death and consequently leading to incomplete neural tube closure through oxidative stress during embryogenesis

**DOI:** 10.1007/s10565-024-09894-1

**Published:** 2024-07-03

**Authors:** Yu Yan, Wenyi Huang, Xiaoting Lu, Xianxian Chen, Yingyi Shan, Xin Luo, Yu Li, Xuesong Yang, Chun Li

**Affiliations:** 1 School of Nursing, Guangzhou University of Chinese Medicine, Guangzhou, 510405 China; 2 Research Center of Integrative Medicine, School Basic Medical Sciences, Guangzhou University of Chinese Medicine, Guangzhou, China; 3 Division of Histology and Embryology, Medical College, Jinan University, Guangzhou, 510632 China; 4 Clinical Research Center, Clifford Hospital, Guangzhou, 511495 China; 5 Department of Endocrinology and Metabolism, Shanghai Sixth People’s Hospital Affiliated to Shanghai Jiao Tong University School of Medicine, Shanghai Clinical Center of Diabetes, Shanghai Key Laboratory of Diabetes Mellitus, Shanghai Key Clinical Center for Metabolic Disease, Shanghai Diabetes Institute, Shanghai, 200233 China; 6 Department of Urology, Sun Yat-Sen University Cancer Center, State Key Laboratory of Oncology in Southern China, Collaborative Innovation Center for Cancer Medicine, Guangdong Provincial Clinical Research Center for Cancer, Guangzhou, 510060 China

**Keywords:** Nanoparticles, Oxidative stress, Ferroptosis, Autophagy, Apoptosis, Neurogenesis

## Abstract

The implementation of Zinc oxide nanoparticles (ZnO NPs) raises concerns regarding their potential toxic effects on human health. Although more and more researches have confirmed the toxic effects of ZnO NPs, limited attention has been given to their impact on the early embryonic nervous system. This study aimed to explore the impact of exposure to ZnO NPs on early neurogenesis and explore its underlying mechanisms. We conducted experiments here to confirm the hypothesis that exposure to ZnO NPs causes neural tube defects in early embryonic development. We first used mouse and chicken embryos to confirm that ZnO NPs and the Zn^2+^ they release are able to penetrate the placental barrier, influence fetal growth and result in incomplete neural tube closure. Using SH-SY5Y cells, we determined that ZnO NPs-induced incomplete neural tube closure was caused by activation of various cell death modes, including ferroptosis, apoptosis and autophagy. Moreover, dissolved Zn^2+^ played a role in triggering widespread cell death. ZnO NPs were accumulated within mitochondria after entering cells, damaging mitochondrial function and resulting in the over production of reactive oxygen species, ultimately inducing cellular oxidative stress. The N-acetylcysteine (NAC) exhibits significant efficacy in mitigating cellular oxidative stress, thereby alleviating the cytotoxicity and neurotoxicity brought about by ZnO NPs. These findings indicated that the exposure of ZnO NPs in early embryonic development can induce cell death through oxidative stress, resulting in a reduced number of cells involved in early neural tube closure and ultimately resulting in incomplete neural tube closure during embryo development. The findings of this study could raise public awareness regarding the potential risks associated with the exposure and use of ZnO NPs in early pregnancy.

**Graphical Abstract:**

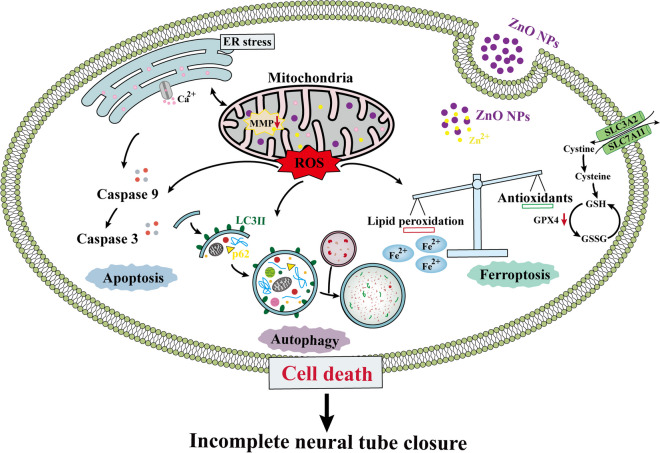

**Supplementary Information:**

The online version contains supplementary material available at 10.1007/s10565-024-09894-1.

## Introduction

Nanomaterials have been applied in various products owing to their unique chemical and physical properties (Xuan et al. 2023). Among the top three nanoparticles in global production, Zinc oxide nanoparticles (ZnO NPs) have gained extensive application in medicine, food processing as well as other industries for their large specific surface area and strong redox ability (Kumar and Dhawan 2013; Nagar et al. 2022; Swain et al. 2016). However, while nanoparticles bring convenience to production and life, they also present substantial health risks. Studies indicated that ZnO NPs can enter the human body through respiration, digestion, skin contact, etc., gather in various organs such as heart, lungs, liver and reproductive system. The accumulation results in serious damage to cells, tissues and organs (Bunderson-Schelvan et al. 2020; Chen et al. 2020; Pei et al. 2023; Staroń et al. 2020). Pregnancy is a special and delicate physiological period. During this period, mother and fetus are vulnerable to the influence of exogenous substances that can lead to adverse pregnancy outcomes. Relevant researches have confirmed that ZnO NPs can reach embryos through the placental barrier, causing fetal developmental abnormalities (Teng et al. 2021; Wang and Wang 2020). Intravenous injection of 20 mg/kg ZnO NPs resulted in an increase in the number of stillbirths and resorbed fetuses and a decrease in embryo weight (Lee et al. 2016). Oral administration of 180 mg/kg ZnO NPs from day 10.5 to day 17.5 of gestation in mice induced placental oxidative stress, cell apoptosis, and resulted in fetal growth restriction and developmental abnormalities (Chen et al. 2020). Oral administration of ZnO NPs to pregnant mice at a higher concentration (300 mg/kg) caused severe placental barrier dysfunction and irreversible fetal developmental toxicity (Teng et al. 2019). Our previous research also proved that exposure to ZnO NPs affects early embryonic nervous system development (Yan et al. 2021). This finding underscores the need for a more comprehensive exploration of the specific mechanisms by which ZnO NPs induce neurotoxicity during embryogenesis.

The nervous system is highly sensitive to the external environment during embryogenesis. Cao’s studies have shown that exposure to carboxylic graphene oxide nanoparticles during embryonic development may induce neurotoxicity and symptoms akin to Parkinson’s disease (Cao et al. 2021). Zhang et al. also confirmed that maternal exposure to AgNPs can disrupt astrocyte morphology and reduce postsynaptic protein expression, leading to neurodegeneration (Zhang et al. 2021). These studies suggested a strong association between exposure to nanoparticles during pregnancy and abnormalities in fetal nervous system development. However, it is worth noting that most of current researches on the neurotoxic effects of nanoparticles focus on late stages rather than early stages of nervous system development. It is known that the development of early nervous system at the gastrula stage is a crucial period during embryogenesis, which marks the beginning of embryonic nervous system development. Afterwards, through the regulation of numerous signaling pathways, neural tube cells undergo neurogenesis and eventually differentiate into complex nervous systems. Disturbances in early nervous system development are likely to cause fetal brain functional development disorders, such as genetic changes and impaired learning and memory abilities (Wang et al. 2022). This study concentrated on the harmful impacts of ZnO NPs on early embryonic nervous system development and explored its potential mechanisms. Our aim is to enhance public awareness, especially among pregnant women, regarding the pathogenicity of ZnO NPs, and to provide an important basis for risk assessment of nanoparticles used in early pregnancy. Our prior researches have shown that exposure of embryos to ZnO NPs in the early stages of neural development can lead to abnormalities of neural crest cells and defects in neural tube closure (Liu et al. 2020; Yan et al. 2021). We have preliminarily confirmed that this phenotype is caused by the activation of apoptosis mediated by endoplasmic reticulum stress after ZnO NPs treatment. In view of the complexity and multifactorial nature of toxicological mechanisms, further exploration is warranted to understand abnormalities of embryonic nervous system induced by ZnO NPs.

The nervous system is the dominant system for physiological functions in the body. Exposure to environmental toxicants can lead to incomplete neural tube closure in early embryogenesis, which is one of the manifestations of neural tube defects. It may evolve into congenital abnormalities of the central nervous system and is the second most prevalent birth defects following congenital heart disease. Examples of neural tube defects include anencephaly, myelomeningocele and other phenotypes. Neural tube formation is closely related to precise temporal and spatial regulation of multiple genes (Heusinkveld et al. 2021; Moon and Xiong 2022). Therefore, the pathogenesis of incomplete neural tube closure also involves the participation of different molecular pathways, such as homocysteine, folic acid metabolism disorder, disruption of cytoskeleton and cell migration ability, as well as cell death (Cao et al. 2022; Hassan et al. 2022; Wang and Ren 2021; Wei et al. 2020). Cell death mechanisms are a significant contributor to neurodevelopmental toxic events and have received considerable attention in recent years. Many studies have confirmed the involvement of programmed cell deaths in neurodegenerative diseases and brain injury, such as autophagy (Cao et al. 2020b; Xu et al. 2019), apoptosis (Cao et al. 2020a; Yang et al. 2013), ferroptosis (Kim et al. 2021). However, there remains a notable research gap concerning the roles of these types of programmed cell death in early embryonic nervous system development. In addition, from a deeper mechanistic perspective, the endogenous antioxidant defense mechanisms of developing nerve cells are more fragile than those of the mature brain, making the early embryonic nervous system more susceptible to oxidative stress. In fact, our prior researches have confirmed that ZnO NPs can cause increased levels of oxidative stress in nerve cells (Liu et al. 2020). In this study, we used well-established animal models that are widely used in toxicity assessment and early neurodevelopment research-chicken embryo (Lecca et al. 2023; Patel et al. 2019). The aim is to explore the comprehensive toxic effects of ZnO NPs exposure on the nervous system at different concentrations during embryogenesis. In addition, we seek to verify whether oxidative stress is the upstream pathway of ZnO NPs-induced cytotoxicity.

## Methods

### Characterization and preparation of ZnO NPs

After the sonication of ZnO NPs, the following experiments were performed to characterize the material properties. The physical appearance and size of ZnO NPs powder (CAS677450-5G, Sigma-Aldrich, USA) was evaluated using the transmission electron microscope (TEM, TECNA12 microscope, FEI, USA) and field emission scanning electron microscope (SEM, ULTRA55 microscope, ZEISS, GER). The zeta potential of ZnO NPs in serum, EC culture (early chick culture) and medium was measured by electrophoretic light scattering (ELS) while the hydrated particle size were assessed by dynamic light scattering (DLS), both with a Zetasizer Nano ZS instrument (Malvern Instruments Ltd, UK). The crystal structure and specific surface area of ZnO NPs were determined by X-ray powder diffractometer (XRD, Thermo Fisher, USA) and Micromeritics ASAP 2010 M + C instrument (Micromeritics Co., GA, USA), respectively. ZnO NPs were prepared into a nanoparticle suspension of the desired concentration using distilled water, physiological saline (0.9%) or Dulbecco’s Modified Eagle’s Medium (DMEM, C11995500BT, Gibco, USA). Ultrasonic vibration was performed with an ultrasonic cleaning instrument (JP-040, CGOLDNWALL, JPN) for 1 h, followed by subsequent animal and cell experiments respectively.

### *Determination of free Zn*^*2*+^*concentration*

Inductively coupled plasma mass spectrometry (ICP-MS, Invitrogen, Germany) was utilized to determine the concentration of Zn^2+^of ICR mice placenta and embryos. Briefly, the tissue was placed in concentrated nitric acid overnight, followed by digestion with 30% hydrogen peroxide at 200 °C. The resulting digestion liquid was cooled and centrifuged at 10,000 rpm for 5 min to collect the supernatant, which was then analyzed using ICP-MS. To assess the solubility of nanoparticles in liquid, ZnO NPs were added to DMEM and subject to sonication for 1 h. The resulting suspension was placed in a 37 °C cell culture incubator. At predetermined time intervals (0, 2, 4, 6, 8, 12, 24 and 48 h), the supernatant was collected after centrifugation at 10,000 rpm for 5 min, and Zn^2+^ concentration was assessed using ICP-MS.

### ZnO NPs exposure animal model

8-weeks-old ICR mice were purchased from Guangdong Medical Laboratory Animal Center (Guangdong, China). The mice were placed in a 21–22 °C environment for a 12-h light/dark cycle and ad libitum access to food and water. Following this, the male and female mice were caged together for 1 day. Pregnancy in the female mice was determined according to the presence of vaginal plugs on the second day’s morning. Pregnant mice were allocated into distinct groups using randomization techniques (N = 6): the saline group, 5 mg/kg, 10 mg/kg and 20 mg/kg ZnO NPs group. Pregnant mice were injected with different concentration of ZnO NPs into the tail vein on embryonic days E5.5, E7.5 and E9.5 respectively. On E10.5, the mice were anesthetized with pentobarbital and subsequently euthanized. The placenta and embryos were harvested for subsequent experiments (Supplementary Fig. 1A).

Fertilized Leghorn eggs were purchased from the South China Agriculture University (Guangdong, China) and were subjected to place in a humidified incubator (Yiheng Instrument, Shanghai, China). The incubator was set to a temperature of 38 °C and a humidity level of 70% until the chicken embryo reached desired stage. It should be emphasized that the developmental stages of chicken embryos are usually delineated according to the Hamburger and Hamilton (HH) staging, which provides a reference and standard for identifying various important stages involved in early neurogenesis of chicken embryo (Bluemel et al. 2021; Misske et al. 2007). Specifically, HH0 is the first few hours of incubation and the embryo at this period is basically fertilized eggs. When incubated to HH4-HH5 (16–22 h), the embryo develops into a gastrula. At this time, the notochord stimulates the ectoderm to differentiate into neuroectoderm and leads to the formation of neural plate. The lateral edges of both ends of the neural plate then gradually rise, forming neural folds when they develop to HH6-HH7 (23–26 h). Afterwards, the neural folds at both ends continue to approach and encounter at the midline, and merge into the neural tube at HH10-HH11 (33–45 h). Therefore, to more clearly observe the effects of ZnO NPs on various important stages of early embryonic neural development. HH0 chicken embryos were subjected to incubated in EC culture at a temperature of 38℃ within a humidified environment, experiments were conducted both with and without the presence of ZnO NPs (12.5, 25 and 50 μg/ml) or 5 mM N-acetylcysteine (NAC) until HH10. We observed chicken embryos in each group at HH0, HH4, HH7 and HH10 and the surviving embryos (N ≥ 12 embryos in each group) were harvested at the HH10 stage for the evaluation of morphology in the developing embryonic neural tubes, including the distance of approaching neural folds, the area of approaching neural folds and the rostral-caudal distance of closing neural folds. The modeling method of chicken embryos exposed to ZnO NPs described above is presented in Supplementary Fig. 1B in the form of a schematic diagram.

### Whole-mount embryos immunofluorescent staining

The immunofluorescent staining was conducted on whole-mount chicken embryos according to established protocols (Song et al. 2020; Yahya et al. 2021). Following incubation, the embryos were collected and preserved in 4% PFA at 4℃ overnight. Subsequently, the fixed chicken embryos were exposed to primary antibodies (Pax7, 1:500, AB528428, DSHB, USA) at 4℃ on a shaker for an overnight incubation. Following thorough rinsing in PBST (0.1% Tween-20), the embryos were incubated with Alexa fluor 555 secondary antibodies (1:1000, Invitrogen, USA) on a shaker overnight at 4℃. DAPI (1:1000, Invitrogen, USA) was used for counterstaining all embryos for an hour and imaged using a Leica M205FA fluorescence stereo microscope.

### RNA sequencing (RNA-seq) transcript profiling

The embryos of HH10 chickens in both the control group and the group treated with 50 µg/ml of ZnO NPs were collected and sequenced by Novogene Bioinformatics Technology Co., Ltd (Guangdong, China) to detect differentially expressed genes (DEGs) between the two groups. DEGs with |log2 fold change (FC)|> 1 and p-value < 0.05 were considered to be significantly differentially expressed genes. The RNA-seq data obtained from this analysis were subsequently submitted to the Gene Expression Omnibus (GEO) database and the GEO number is GSE121507. The raw image data files acquired from high-throughput sequencing were processed using CASAVA base identification analysis to generate original sequencing sequences. The outcomes were saved in FASTQ file format, encompassing the sequence data (reads) and associated sequencing quality information. Rigorous quality control measures were implemented on the sequencing data, ensuring a sequencing error rate of less than 1% per base position and removing adapters and low-quality reads. After reference sequence comparison analysis, gene expression level analysis and overall quality assessment, the DEGs between the two groups were obtained, and then heatmap and GO enrichment analysis were generated using the Seurat R package (version 5.0.1), and protein–protein interaction (PPI) analysis was performed using the STRING software.

### Cell culture

We used SH-SY5Y cells (ATCC, USA) as a model for in vitro experiments. SH-SY5Y cells are considered an ideal in vitro model for neurotoxicology studies because of their neurogenic characteristics and ability to differentiate into neuron-like cells (Hoffmann et al. 2023; Lopez-Suarez et al. 2022). Cells were maintained in DMEM containing 10% fetal bovine serum (16410071, Gibco, USA), 100units/ml penicillin and 100 μg/ml streptomycin (15140122, Gibco, USA) in the incubator set at 37℃ with a 5% CO_2_ concentration and passaged every two days.

### Cell viability test

Cell viability was evaluated utilizing Cell Counting Kit-8 (CCK8, ab228554, Abcam, UK) following established protocols (Liu et al. 2020). Briefly, cells were plated in 96-well dishes (1.5 × 10^3^ cells/cm^2^) and were treated with or without the presence of 300 µM ZnCl_2_, ZnO NPs (2.5, 5, 12.5, 25 and 50 µg/ml), deferoxamine (DFO, 1, 5, 10, 50, 100, 200 µM) or NAC (0.5, 1, 2, 5, 10 and 20 mM). After incubation (2, 4 and 6 h), 10µL of CCK-8 working reagent (5 g/L) was added to the wells of the 96-well dishes and incubated for an additional 4 h at 37℃. Absorbance values were taken at a wavelength of 450 nm utilizing the Bio-Rad 450 microplate reader (Bio-Rad, CA, USA).

### Transmission *electron* microscopy of SH-SY5Y cells

SH-SY5Y cells were cultured in 25cm^2^ cell culture flasks at a concentration of 8 × 10^4^ cells/cm^2^. Cells were cultured with DMEM with or without the presence of ZnO NPs (12.5, 25 and 50 μg/ml) for 6 h after the cell density reached 80%. Subsequently, the cells in each group were subjected to three washes with PBS, followed by digestion with trypsin and centrifugation at 1,000 rpm for 5 min. The supernatant was then discarded to collect cells (about the size of half a mung bean). Cells were fixed in 0.1 mol/L PBS containing 2.5% glutaraldehyde for 2 h, and then dehydrated, embedded, sectioned, and stained in the same manner as in vivo experiments. Subsequently, the cell mitochondria alteration and autophagosome were examined using TEM.

### Cell immunofluorescent staining

Cell immunostaining was performed as previous reported (Yan et al. 2021). Briefly, cells were cultured in 6-well dishes (5 × 10^3^ cells/cm^2^) and were subjected to the treatment with or without the presence of 300 µM ZnCl_2_ or ZnO NPs (12.5, 25 and 50 µg/ml) for 6 h. Cells were then harvested and fixed overnight in 4% PFA at 4℃. Cells were subjected to immunostaining using specific antibodies: LC3 (Microtubule-associated protein 1A/1B-light chain 3, 1:500, ab192890, Abcam, UK), p62 (sequestosome 1, 1:500, AP2183B, Abcepta, USA), GPX4 (Glutathione peroxidase 4, 1:200, DF6701, Affinity biosciences, China), FTL (Ferritin light chain, 1:200, DF6604, Affinity biosciences, China), SLC7A11 (Solute carrier family 7 member 11, 1:200, DF12509, Affinity biosciences, China). In summary, cells were subjected to overnight incubation with primary antibodies at 4℃ on a shaker, followed by thorough rinsing in PBST (0.1% Tween-20). Subsequently, the cells underwent overnight incubation with Alexa fluor 555 or 488 secondary antibodies (1:1000, Invitrogen, USA) at 4℃ on a shaker. The cells were then stained with DAPI (1:1000, Invitrogen, USA) for an hour before being imaged using an Olympus IX51 inverted fluorescent microscope.

#### Flow cytometry-based detection of apoptosis

To assess apoptosis rates and ROS levels in SH-SY5Y cells, we used an Annexin V-FITC apoptosis kit (88–8005-72, Thermo Fisher, USA) conducted the analysis through flow cytometry. The cells (8 × 10^4^ cells/cm^2^) were inoculated into 6-well dishes overnight and then exposed to 300 µM ZnCl_2_, ZnO NPs (12.5, 25 and 50 μg/ml) or 5 mM NAC for 2, 4 or 6 h. Subsequently, the cells were digested with trypsin and centrifuged at 1,000 rpm for 5 min, 5μL annexin V-FITC and 5μL PI (50 μg/mL) were included to mix with cells. After vortex, the cells were subjected to incubation at room temperature for 20 min and analyzed utilizing a FACS-can flow cytometer (Becton–Dickinson, San Jose, USA). Flow cytometry detection results are presented as fluorescence intensity scatter plots: the lower left quadrant, lower right quadrant, upper right quadrant and upper left quadrant are viable cells, early apoptotic cells, late apoptotic cells and necrotic cells respectively.

#### Reactive oxygen species (ROS) detection

The cells were plated in 6-well dishes (8 × 10^4^ cells/cm^2^) and exposed to 300 µM ZnCl_2_, ZnO NPs (12.5, 25 and 50 μg/ml) or 5 mM NAC for 6 h. Subsequently, the cells were suspended in ROS working reagent (S0033M, Beyotime, China) and stained at 37℃ for 30 min. Following a PBS wash, the cells were re-suspended in 500μL PBS and the ROS levels were quantified using a FACS-can flow cytometer. Cells were inoculated into confocal dishes (8 × 10^4^ cells/cm^2^) and incubated with DMEM containing 300 µM ZnCl_2_, varying concentrations of ZnO NPs (12.5, 25 and 50 μg/ml) or 5 mM NAC for 6 h. Subsequently, the initial medium was removed and replaced with 1 mL ROS working solution, which was then incubated for an additional 10 min at 37℃. Subsequently, the cells were visualized using an Olympus IX51 inverted fluorescent microscope.

#### Assay of mitochondrial membrane potential with JC-1 assay kit

The cells were plated in 6-well dishes (5 × 10^3^ cells/cm^2^) and exposed to 300 µM ZnCl_2_ or ZnO NPs (12.5, 25 and 50 μg/ml). The supernatant was discarded, and the cells were incubated with DMEM containing 10 μg/ml JC-1 working reagent (C2005, Beyotime, China) at 37℃ for 30 min and then washed three times with PBS. The images were acquired within 30 min using an Olympus IX51 inverted fluorescent microscope.

#### *Assay of Fe*^*2*+^*contents with FerroOrange*

The cells were plated in confocal dishes at a concentration of 5 × 10^3^ cells/cm^2^ as described above and exposed to 300 µM ZnCl_2_, varying concentrations of ZnO NPs (12.5, 25 and 50 μg/ml) or 5 mM NAC for 6 h. Fe^2+^ contents were detected using FerroOrange (F374, Dojindo, Japan). After removing the supernatant cells were stained with DMEM containing 1 μM FerroOrange at 37℃ for 30 min and then rinsed three times with PBS. The images were acquired within 30 min using an Olympus IX51 inverted fluorescent microscope.

#### Calcein acetoxymethyl ester/propidium iodide (Calcein-AM/PI) staining

The cells were plated in confocal dishes (8 × 10^4^ cells/cm^2^) and exposed to 300 µM ZnCl_2_, varying concentrations of ZnO NPs (12.5, 25 and 50 μg/ml) or 5 mM NAC. After labeling with Calcein-AM/PI dye (E-CK-A354, Elabscience, China) at 37℃ for 30 min, the living cells (green fluorescence) and dead cells (red fluorescence) were imaged using an Olympus IX51 inverted fluorescent microscope.

#### Total superoxide dismutase (SOD) activity

Cells were plated in 6-well dishes (8 × 10^4^ cells/cm^2^) and then treated with or without varying concentrations of ZnO NPs (12.5, 25 and 50 μg/ml) for 6 h. Subsequently, the cells were homogenated with 100μL PBS and then centrifuged at 1,000 rpm for 5 min. After adding the SOD assay working solution (S0109, Beyotime, China) and reaction initiation solution to the supernatant, the liquid was incubated at 37℃ for 30 min. The absorbance values were quantified at a wavelength of 450 nm utilizing the microplate reader.

#### Lipid peroxidation malondialdehyde (MDA) concentration

The MDA concentration of cells was evaluated using the Lipid Peroxidation MDA Assay Kit (S0131S, Beyotime, China). Briefly, the cells were plated in 6-well dishes (8 × 10^4^ cells/cm^2^), followed by treatment with or without varying concentrations of ZnO NPs (12.5, 25 and 50 μg/ml) for 6 h. Subsequently, the cells were homogenated with 100μL PBS and then centrifuged at 1,000 rpm for 5 min. After adding the MDA assay working solution to the supernatant, the liquid was incubated at 100℃ for 15 min. After being cooled to room temperature, the liquid was centrifuged (1000 g) for 10 min. The absorbance values were recorded at a wavelength of 532 nm utilizing the microplate reader.

#### Glutathione (GSH) concentration

The GSH concentration of cells were evaluated using the GSH and GSSG Assay Kit (S0053, Beyotime, China). Cells were plated in 6-well dishes (8 × 10^4^ cells/cm^2^), followed by treatment with or without varying concentrations of ZnO NPs (12.5, 25 and 50 μg/ml) for 6 h. Subsequently, the cells were homogenated with protein removal reagent M solution and then centrifuged at 1,000 g for 10 min. The GSH assay working reagent was added to the supernatant and incubated for 5 min. Subsequently, the NAPDH working solution was added, and the mixture was further incubated for 30 min. The absorbance values were recorded at a wavelength of 412 nm utilizing the microplate reader.

#### Western blotting analysis

The protein extraction and immunoblotting techniques utilized in this study were performed as previous reported (Yan et al. 2021). Cell collection and lysis were performed using CytoBuste protein extraction reagent (#71,009, Novagen, Germany) supplemented with protease and phosphatase inhibitors (Roche, Switzerland). Total protein concentrations were determined using a BCA quantification kit (BCA01, DingGuo BioTECH, China). The protein loading amount was 30 μg/sample and protein were separated by SDS-PAGE and subsequently transferred to PVDF membranes (Bio-Rad). The membranes were obstructed with 5% Difco skim milk (#232,100, BD, USA) and then exposed to primary antibody (FTL) for 18 h on a shaker at 4 °C, followed by incubation with secondary antibody (7074P2, Cell Signaling Technology, USA) for 1 h at room temperature. Subsequently, the bands corresponding to the proteins of interest were detected utilizing an ECL kit (#34,079, Thermo Fisher, USA) and GeneGnome5 system (SYNGENE, China). The intensity of the bands was assessed using Quantity One software (Bio-Rad, USA). FTL was diluted 1000-fold in 5% skim milk, and the secondary antibodies were diluted 3000-fold. At minimum of three separate experiments were conducted.

#### Data analysis

The data were statistically analyzed using IBM SPSS statistics 26.0 and graphed using GraphPad Prism 8.0. Experiments were conducted independently at least three times. The Shapiro–Wilk test was used to assess the normality of all data in this study. If the data were normally distributed, they were presented as mean ± SEM. Multiple comparisons between groups were made using the LSD test in one-way ANOVA for data with homogeneity of variances. For data with heterogeneity of variances, Dunnett´s T3 test was employed to compare between groups. The nonparametric Kruskal–Wallis rank sum test was utilized to analyze data of Non-Normal distribution. The chi-squared test was employed to assess the differences in rates among the experimental groups. The chi-squared test was employed to assess the differences in rates among the experimental groups. Statistical significance was established at P < 0.05, while highly significant differences were indicated at P < 0.01 and P < 0.001 between control and experimental groups.

## Results

### The physical and chemical properties of ZnO NPs

**Fig. 1:**
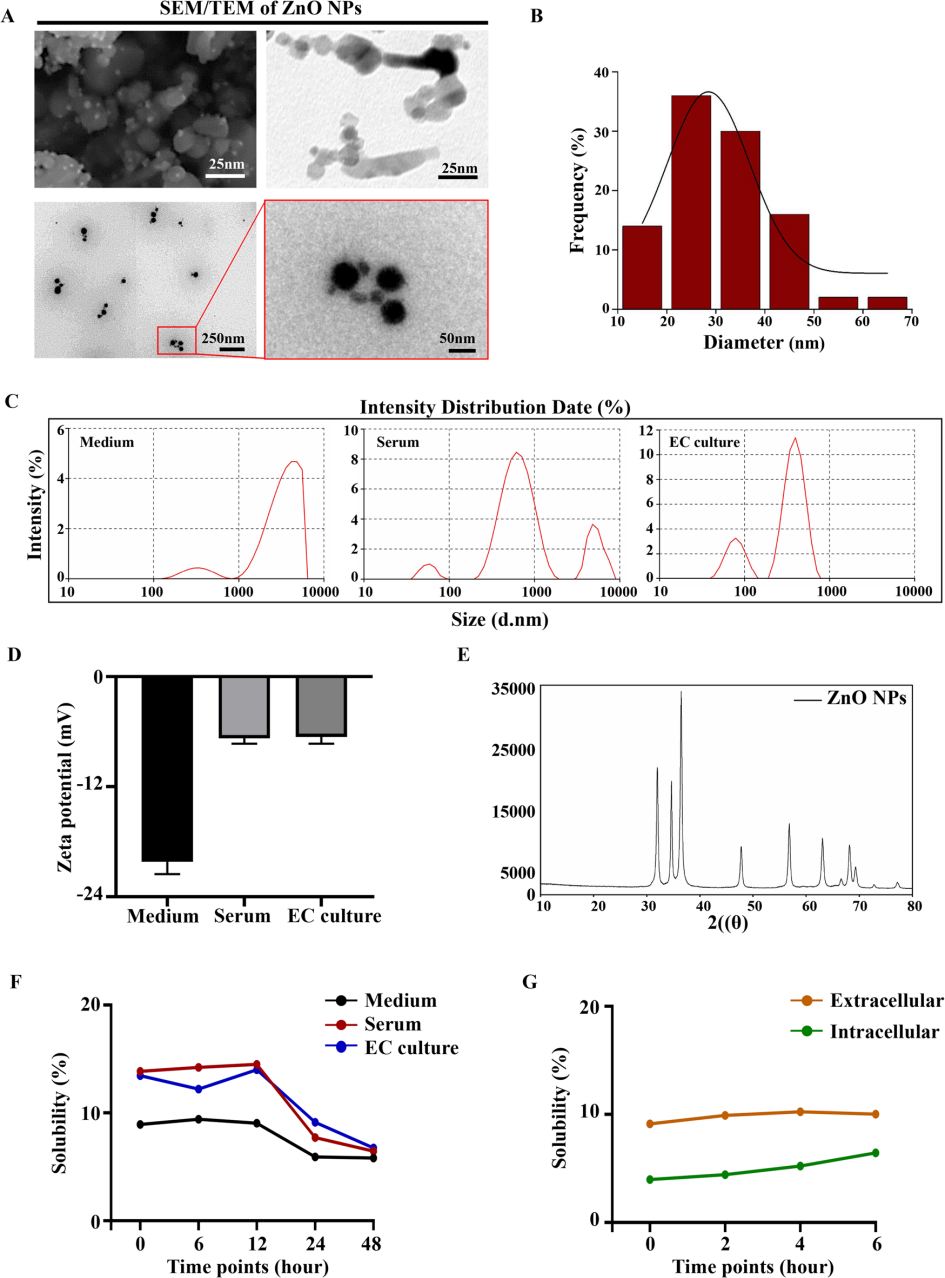
**Physical and chemical characterization of ZnO NPs** (**A**) The representative SEM and TEM images of ZnO NPs. (**B**) The particle size distribution histogram determined from TEM images. (**C-D**) Particles size distribution and zeta potential measurements of ZnO NPs in different culture media. Measurements were performed in triplicate and the average value obtained was calculated by the software provided with instrument. (**E**) XRD patterns of ZnO NPs. (**F**) The solubility of ZnO NPs dissolved in different culture media at different time points. (**G**) The extracellular and intracellular solubility of ZnO NPs at different time points

The first step in investigating the toxic impacts of commercially accessible ZnO NPs involves evaluating the characteristics. Results of SEM and TEM analysis showed that the morphology of ZnO NPs is mainly spherical or rod-shaped (Fig. 1A), with a size range of 11-64nm and an average particle size of 31.71 ± 1.672nm (Fig. 1B). In addition, the surface area of ZnO NPs is 26.069 m^2^ g^−1^ and the polydispersity index (PDI) is 0.15 ± 0.02. Considering that this study used multiple models, the hydrated particle size and zeta potential of ZnO NPs were characterized in medium, serum and EC culture (Fig. 1C-D). The potential value is lower than 30mV, indicating that ZnO NPs are easy to agglomerate in solution (Wu et al. 2020). Therefore, we fully ultrasonically dispersion treatment on the ZnO NPs suspension before subsequent in vivo and in vitro tests to ensure uniform exposure of the ZnO NPs, prevent high concentration aggregation effects, and improve the dosage accuracy of toxicological studies. In addition, the XRD pattern of ZnO NPs is consistent with the standard data of zinc and oxygen, indicating that ZnO NPs have high purity (Fig. 1E). The solubility of ZNO NPs in different culture media was less than 15% (Fig. 1F), and the extracellular solubility of ZNO NPs is greater than that of intracellular solubility (Fig. 1G).

### *ZnO NPs and the Zn*^*2*+^*they release penetrate placental barrier of mouse and cause abnormal development during chicken embryogenesis*

**Fig. 2:**
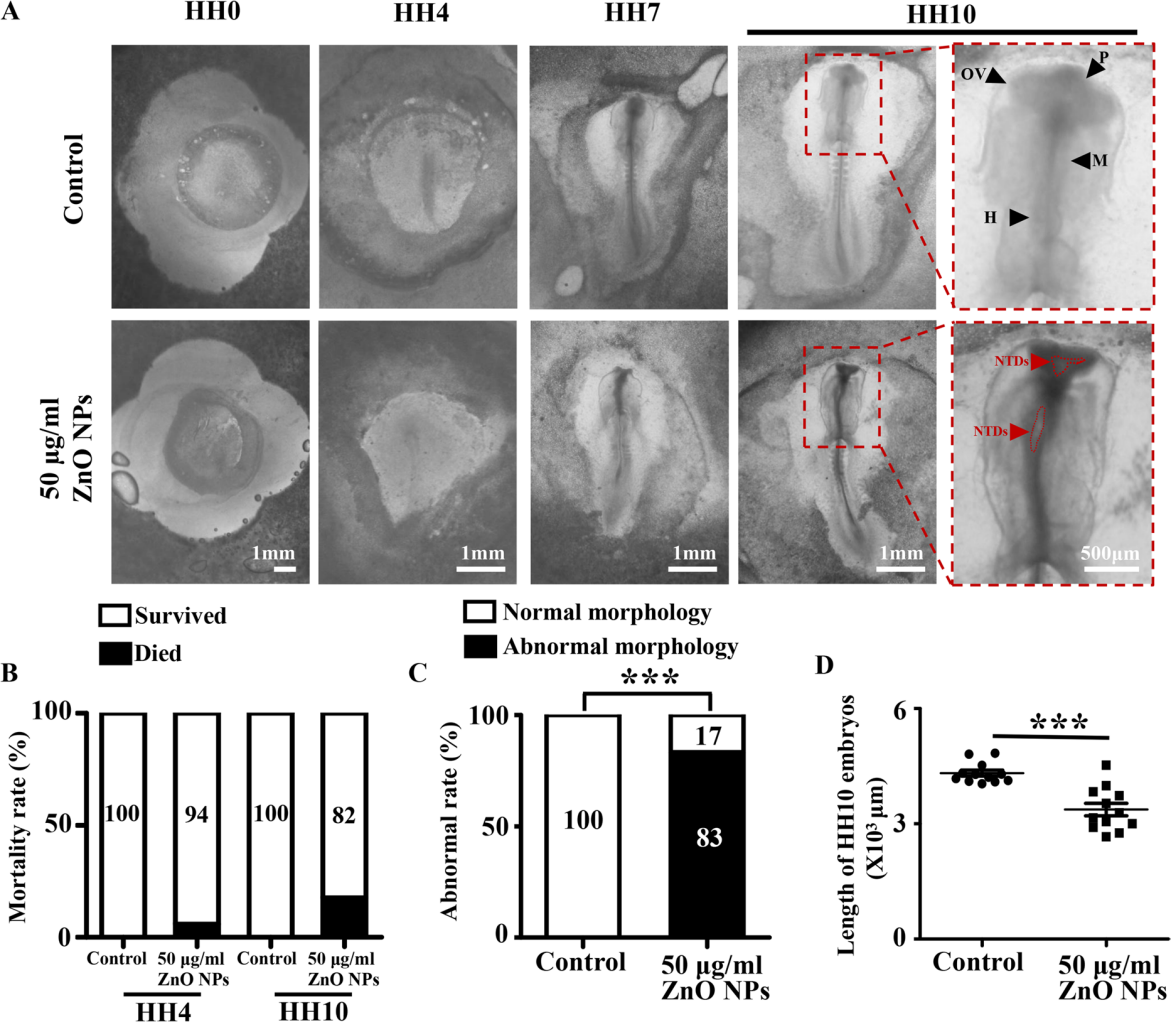
**Assessment of the effect of ZnO NPs exposure during embryogenesis** (**A**) Bright-field images of HH10 chicken embryos in each group. The black arrows indicate morphology of the neural tube of HH10 chicken embryos and the red arrow indicate the abnormal morphology of the neural tube of HH10 chicken embryos. (**B-C**) Bar charts showing the mortality rate and abnormal rate of HH10 chicken embryos in each group. (**D**) Scatter plot showing the length of HH10 chicken embryos in each group. OV, optic vesicle; P, forebrain; M, midbrain; R, rhombomere. *P < 0.05, **P < 0.01, ***P < 0.001

To determine whether ZnO NPs can cross the placental barrier, we injected different concentrations of ZnO NPs into pregnant mice every other day starting from E5.5 by tail vein injection, and harvested the placenta and embryos on E10.5 for evaluation. The results showed that the concentration of free Zn^2+^ in the placenta and embryos of pregnant mice exposed to ZnO NPs increased significantly. Moreover, this increase exhibited a dose-dependent trend (Supplementary Fig. 2A-B), suggesting that ZnO NPs and the Zn^2+^ they release penetrate the placental barrier and further damage the early embryonic development. Next, we used the chicken embryo model to further explore the adverse impacts of ZnO NPs on early embryos. The chicken embryos were extracted after fertilization and subsequently placed in culture media containing varying concentrations of ZnO NPs for in vitro cultivation. The embryos were cultured until they developed to the HH10 stage, and then they were harvested for further observation. Chicken embryos treated with ZnO NPs showed varying degrees of developmental abnormalities, including increased abnormal rates and restricted embryonic growth (Fig. 2A-D). The above results confirmed that ZnO NPs have toxic effects on early embryonic development.

### ZnO NPs cause abnormal development of the nervous system during early chicken embryogenesis

**Fig. 3:**
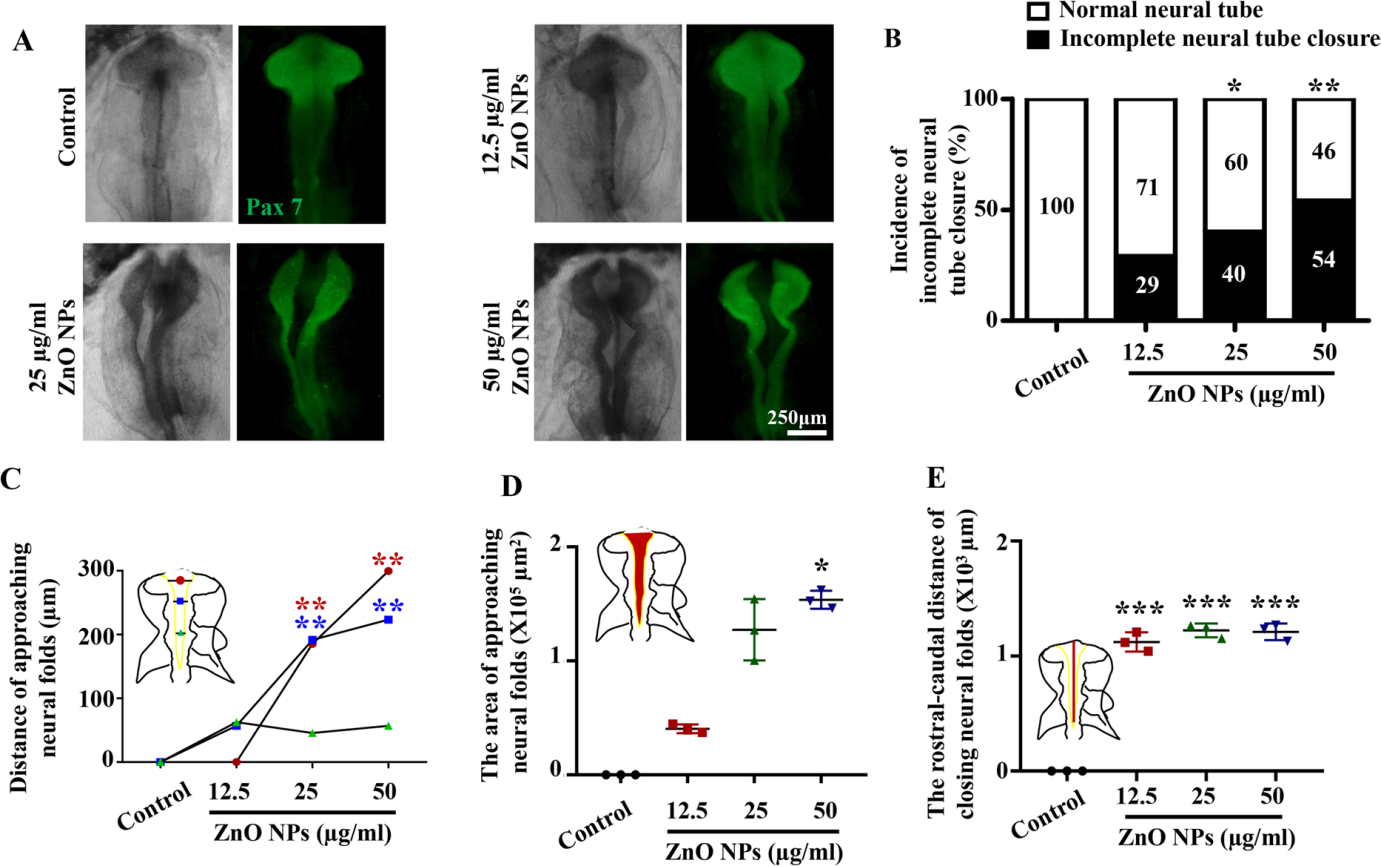
**Assessment of the nervous system development of chicken embryos exposed to different concentrations of ZnO NPs** (**A**) Representative images of chick embryo neural tubes in the control group and groups treated with various concentrations of ZnO NPs. (**B**) Bar chart showing the incidence of incomplete neural tube closure of HH10 chicken embryos in the control group and groups treated with various concentrations of ZnO NPs. (**C**) Bar chart showing the distance of approaching neural folds in each group (the red circles represent the distance of approaching neural folds in cross-section of the forebrain; the blue squares represent the distance of approaching neural folds in cross-section of the midbrain; the green triangles represent the distance of approaching neural folds in cross-section of the hindbrain). (**D**) Bar chart showing the area of approaching neural folds in each group. (**E**) Bar chart showing the rostral-caudal distance of closing neural folds in each group. *P < 0.05, **P < 0.01, ***P < 0.001

Our previous studies have shown that the early embryonic nervous system is the main teratogenic target organ of ZnO NPs (Yan et al. 2021). In this study, we used early chicken embryos to further analyze and evaluate the neurotoxic effects of ZnO NPs. Pax7 was used to visualize the neural tube morphology of HH10 chicken embryos in each group (Monsoro-Burq 2015; Wang et al. 2020). The results showed that the risk of incomplete neural tube closure in chicken embryos increased as the concentration of ZnO NPs treatment increased (Figs. 3A-B). Subsequently, a meticulous quantitative analysis was conducted to examine the observed incomplete neural tube closure, thereby confirming that treatment with ZnO NPs primarily resulted in the following manifestations: increased distance of approaching neural folds (Fig. 3C), expanded area of approaching neural folds (Fig. 3D), and increased rostral-caudal distance of closing neural folds (Fig. 3E). These results provided robust data support that ZnO NPs can induce incomplete neural tube closure in early embryos.

### ZnO NPs caused multiple cell death in SH-SY5Y cells

**Fig. 4:**
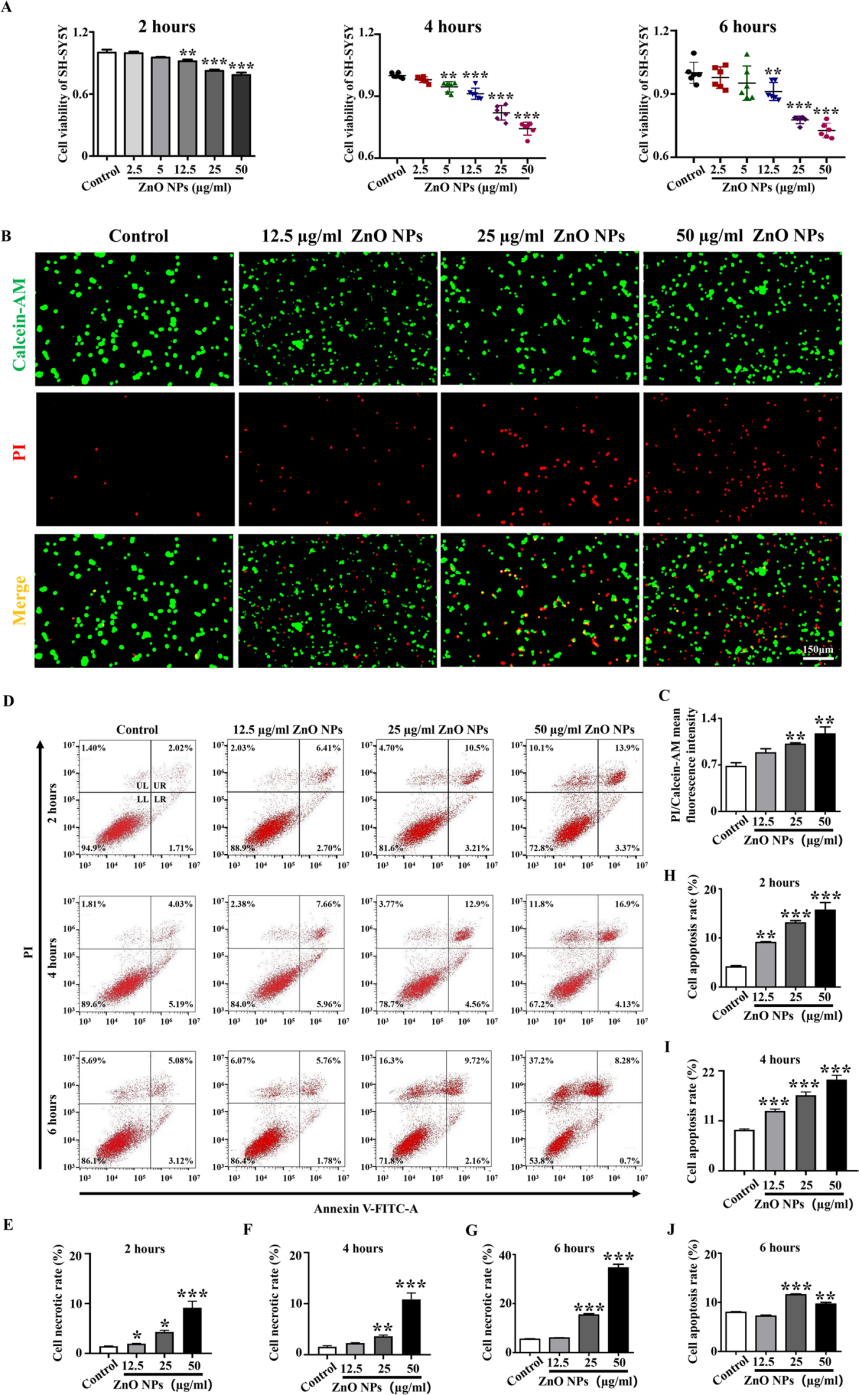
**Assessment of cell viability and cell apoptosis in vitro after different concentrations of ZnO NPs treatment** (**A**) Viability of SH-SY5Y cells after exposure to different concentrations of ZnO NPs for 2, 4 and 6 h. (**B**) Representative images of Calcein-AM/PI double staining of SH-SY5Y cells treated with different concentrations of ZnO NPs for 6 h. (**C**) Bar chart showing mean fluorescence intensity of Calcein-AM/PI in the control and different concentrations of ZnO NPs-treated groups. (**D**) Flow cytometry analysis of the apoptosis of SH-SY5Y cells after exposure to different concentrations of ZnO NPs for 2, 4 and 6 h (LL: viable cells; LR: early apoptotic cells; UR: late apoptotic cells; UL: necrotic cells). (**E–G**) Bar charts showing the necrotic rate of SH-SY5Y cells after exposure to different concentrations of ZnO NPs for 2, 4 and 6 h. (**H-J**) Bar charts showing the apoptosis rate of SH-SY5Y cells after exposure to different concentrations of ZnO NPs for 2, 4 and 6 h. LL, the lower left quadrant; LR, lower right quadrant; UR, upper right quadrant; UL, necrotic cells. *P < 0.05, **P < 0.01, ***P < 0.001

Our previous studies have shown that ZnO NPs induced incomplete neural tube closure in early embryos by causing cell apoptosis (Yan et al. 2021). The cell activity test results in this study demonstrated a significant decrease in cell viability of SH-SY5Y cells when exposed to varying concentrations of ZnO NPs. This decline in cell viability is observed to be dependent on both concentration and duration of exposure, thereby suggesting that exposure to ZnO NPs increases the risk of cell death (Fig. 4A). It is well known that cell death is a genetically controlled process that occurs in multicellular organisms and it includes various modalities such as apoptosis, programmed necrosis, autophagy, and ferroptosis (Moujalled et al. 2021). To further clarify which cell death modalities plays a role in the cytotoxicity of ZnO NPs, different cell death markers and detection methods were utilized to examine the levels of apoptosis, autophagy and ferroptosis. First, we utilized Calcein-AM/PI double staining to label normal cells and apoptotic cells in each group. By measuring the ratio between the mean fluorescence intensity of PI-positive cells and Calcein-AM-positive cells, we found that the cell apoptosis rate was increased in a concentration-dependent manner after ZnO NPs treatment (Fig. 4B-C). Annexin V-FITC/PI staining allowed us to confirm the stages of apoptosis and the results of flow cytometry confirmed that SH-SY5Y cells gradually transitioned from early apoptosis to late apoptosis and even cell necrosis with increasing concentration of ZnO NPs, supporting that ZnO NPs triggered cell apoptosis (Fig. 4D-J).

LC3 (a crucial component for autophagosome formation) is essential for autophagosome synthesis and serves as a biomarker of autophagy initiation (Lin et al. 2021). p62 is an autophagy substrate that is used as a marker of autophagy activity (Ueno and Komatsu 2020). The results of immunofluorescence staining revealed that the expression level of LC3 was up-regulated in SH-SY5Y cells treated with ZnO NPs, while the expression of p62 in the cells was down-regulated, suggesting that ZnO NPs activate autophagy (Supplementary Fig. 3A). In addition, TEM showed that distinct autophagosomes could be observed in SH-SY5Y cells treated with ZnO NPs, which provided additional visual evidence supporting the activation of autophagy through ZnO NPs (Supplementary Fig. 3B).

**Fig. 5:**
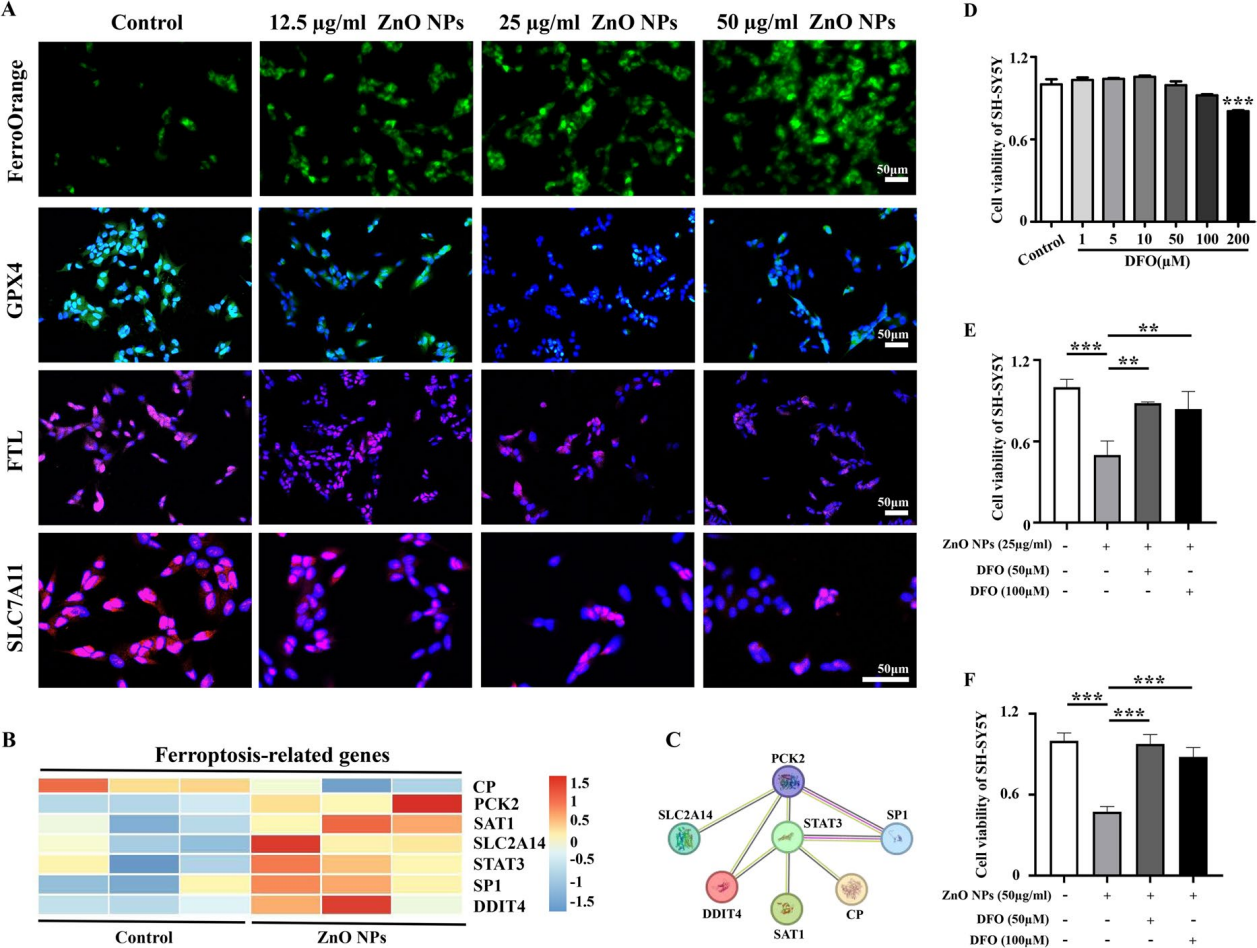
**Assessment of ferroptosis of SH-SY5Y cells after different concentrations of ZnO NPs treatment** (**A**) Representative images of FerroOrange, GPX4, FTL or SLC7A11 immunofluorescence staining of SH-SY5Y cells treated with different concentrations of ZnO NPs for 6 h. (**B**) Heatmap analysis of ferroptosis-related genes expression profiles in HH10 chicken embryos from control and ZnO NPs-treated group. (**C**) PPI network analysis of ferroptosis-related genes in heatmap. (**D**) The viability of SH-SY5Y cells after exposure to various concentrations of DFO for 6 h. (**E–F**) The viability of SH-SY5Y cells after exposure to various concentrations of ZnO NPs or/and DFO for 6 h. *P < 0.05, **P < 0.01, ***P < 0.001

FerroOrange is a novel fluorescent sensor that allows for real-time fluorescent visualization of intracellular Fe^2+^ (Li et al. 2021). In this study, we first used FerroOrange to label intracellular Fe^2+^ and the results revealed that the content of intracellular Fe^2+^ showed a significant increase in SH-SY5Y cells treated with ZnO NPs. Immunofluorescence staining results revealed that the expression of key negative regulators of ferroptosis (GPX4, FTL and SLC7A11) in SH-SY5Y cells were significantly decreased with the increasing concentrations of ZnO NPs (Fig. 5A). Additionally, many proteins are involved in the ferroptosis process, such as the ferroptosis negative-regulator protein ceruloplasmin (CP) and the ferroptosis positive-regulator proteins Phosphoenolpyruvate carboxykinase 2 (PCK2), spermidine/spermine N1-acetyltransferase 1 (SAT1), functional characterization of glucose transporter (SLC2A14), signal transducer and activator of transcription 3 (STAT3), Specificity protein 1 (SP1) and DNA damage-inducible transcript 4 (DDIT4). Among them, CP plays an important role as a ferrous oxidase in iron metabolism and redox reactions and regulate intracellular iron homeostasis (Tang et al. 2021). PCK2 can inhibit cellular ferroptosis by regulating mitochondrial respiration and maintaining glutathione reduction balance (Bluemel et al. 2021; Vinik et al. 2024). SAT1 is a target gene of tumor protein p53 and participates in regulating cell ferroptosis by mediating the expression of ALOX15 (Tang et al. 2021). Overexpression of SLC2A14 leads to excessive glucose uptake by cells, causing oxidative stress and reducing cellular tolerance to iron and promoting ferroptosis. STAT3 activates lysosomal death by mediating cathepsin B overexpression and thereby activates ferroptosis (Gao et al. 2018). SP1 regulates ferroptosis sensitivity in a transcription-dependent or -independent manner (Lyu and Li 2023). DDIT4 can suppress the mTORC1 complex, thereby enhancing autophagy and promoting ferroptosis (Peng et al. 2022). We used RNA-seq analysis to confirm that the expression of the above ferroptosis-related proteins in HH10 chicken embryos was affected by ZnO NPs (Fig. 5B) and intuitively reflected the interaction network between these proteins through PPI analysis (Fig. 5C). To further verify the aforementioned findings, we used the ferroptosis inhibitor DFO to block ferroptosis, confirming the key role of ferroptosis during ZnO NPs-induced cell death. CCK8 data showed that the addition of DFO effectively reverse the decline in cell viability caused by ZnO NPs (Fig. 5D-F). The above results indicated that ZnO NPs induce different types of programmed cell death, resulting in a significant decrease in the number of neuroepithelial cells and contributing to abnormal morphology development of the early neural tube development.

### *Zn*^*2*+^*were partially involved in ZnO NPs-induced cytotoxicity in SH-SY5Y cells*

**Fig. 6:**
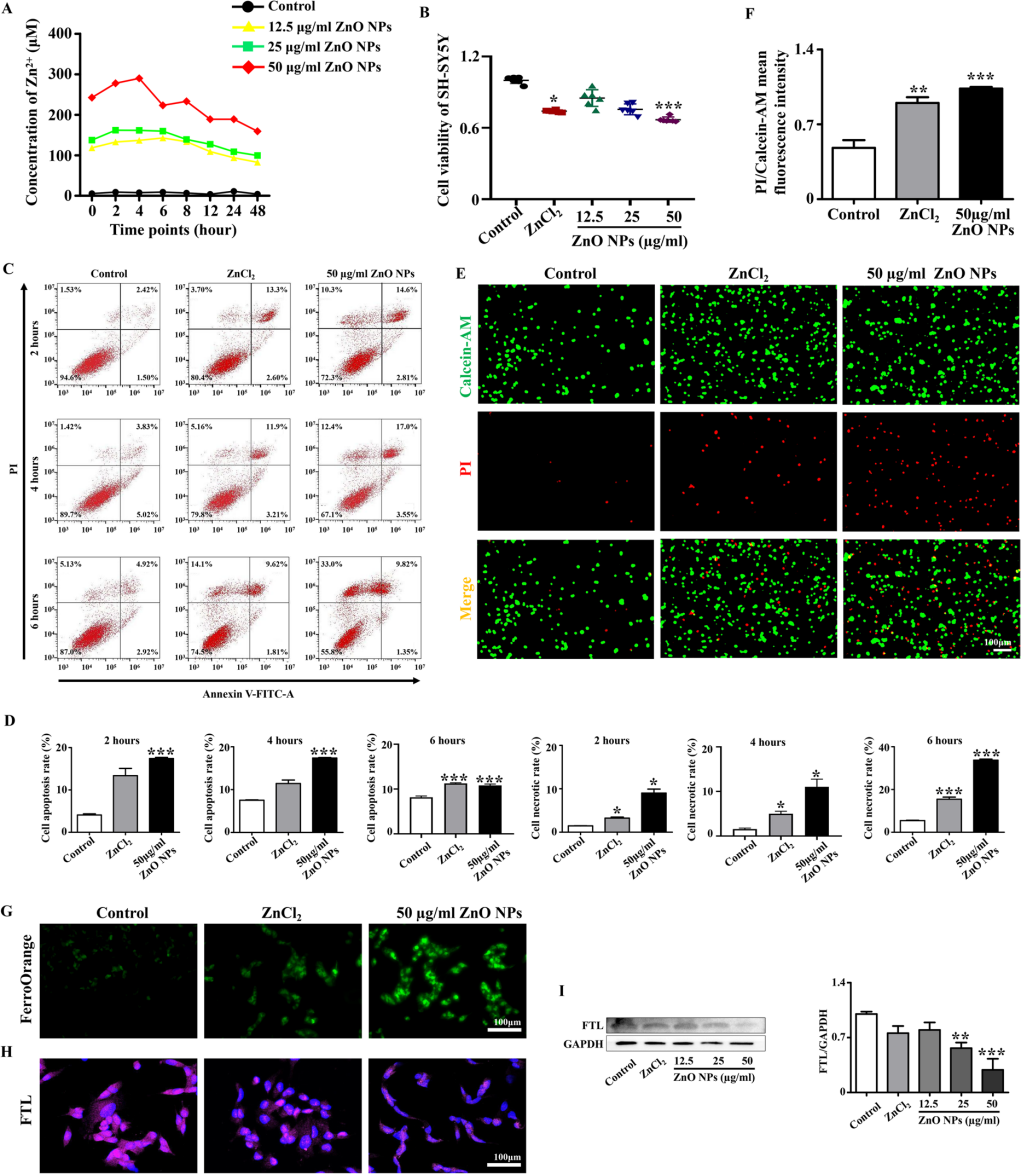
**Assessment of cytotoxic effects of Zn**^**2+**^** on SH-SY5Y cells** (**A**) ICP-MS was used to detect the concentration of Zn^2+^ released from ZnO NPs (12.5 μg/ml, 25 μg/ml and 50 μg/ml) dissolved in DMEM at different time points. (**B**) Viability of SH-SY5Y cells after exposure to 300 μM Zn^2+^ or different concentrations of ZnO NPs for 6 h. (**C**) Flow cytometry analysis of the apoptosis of SH-SY5Y cells after exposure to 300 μM Zn^2+^ or 50 μg/ml ZnO NPs for 2, 4 and 6 h. (**D**) Bar charts showing the apoptosis rate or necrotic rate of SH-SY5Y cells after exposure to 300 μM Zn^2+^ or 50 μg/ml ZnO NPs for 2, 4 and 6 h. (**E**) Representative images of Calcein-AM/PI double staining of SH-SY5Y cells treated with 300 μM Zn^2+^ or 50 μg/ml ZnO NPs for 6 h. (**F**) Bar chart showing mean fluorescence intensity of Calcein-AM/PI of SH-SY5Y cells treated with 300 μM Zn^2+^ or 50 μg/ml ZnO NPs for 6 h. (**G**) Representative images of FerroOrange staining of SH-SY5Y cells treated with 300 μM Zn^2+^ or 50 μg/ml ZnO NPs for 6 h. (**H**) Representative immunofluorescence staining images of FTL of SH-SY5Y cells treated with 300 μM Zn^2+^ or 50 μg/ml ZnO NPs for 6 h. (**I**) Western blot images and bar chart showing the expression of FTL of SH-SY5Y cells treated with 300 μM Zn^2+^ or 50 μg/ml ZnO NPs for 6 h. *P < 0.05, **P < 0.01, ***P < 0.001

ZnO NPs are partially dissolved into Zn^2+^ in solution, and the degree of solubility is related to the solvent and the characteristics of nanoparticles (Cardoso et al. 2021). Initially, we used ICP-MS to clarify the degree of solubility of the ZnO NPs in DMEM. Subsequently, based on the experimental data, we selected 300µM Zn^2+^ to treat SH-SY5Y cells, aiming to confirm the involvement of Zn^2+^ in the ZnO NPs-induced cytotoxicity (Fig. 6A). CCK8 results showed that the viability of SH-SY5Y cells was suppressed after treatment with 300µM Zn^2+^ for 6 h (Fig. 6B). Annexin V-FITC/PI staining based on flow cytometry and Calcein-AM/PI double staining results revealed that cell apoptosis and cell necrosis was activated upon treatment with 300µM Zn^2+^ (Fig. 6C-F). In addition, the results of intracellular iron measurement FerroOrange showed that the intracellular Fe^2+^ content increased after 6 h of treatment with 300µM Zn^2+^ (Fig. 6G). Immunofluorescence staining and western blot data of the ferroptosis biomarker FTL further verified that 300µM Zn^2+^ caused the activation of cellular ferroptosis (Fig. 6H-I). The above results showed that Zn^2+^ plays an important role in the process of ZnO NPs inducing multiple cell death modalities. However, the extensive cell death caused by 300µM Zn^2+^ is not as obvious and severe as that of 50µg/ml ZnO NPs, suggesting that cell death caused by ZnO NPs is due to both the nanoparticles themselves and their dissolved Zn^2+^.

### Oxidative stress is the key upstream mechanism of ZnO NPs-induced cytotoxicity in SH-SY5Y cells

**Fig. 7:**
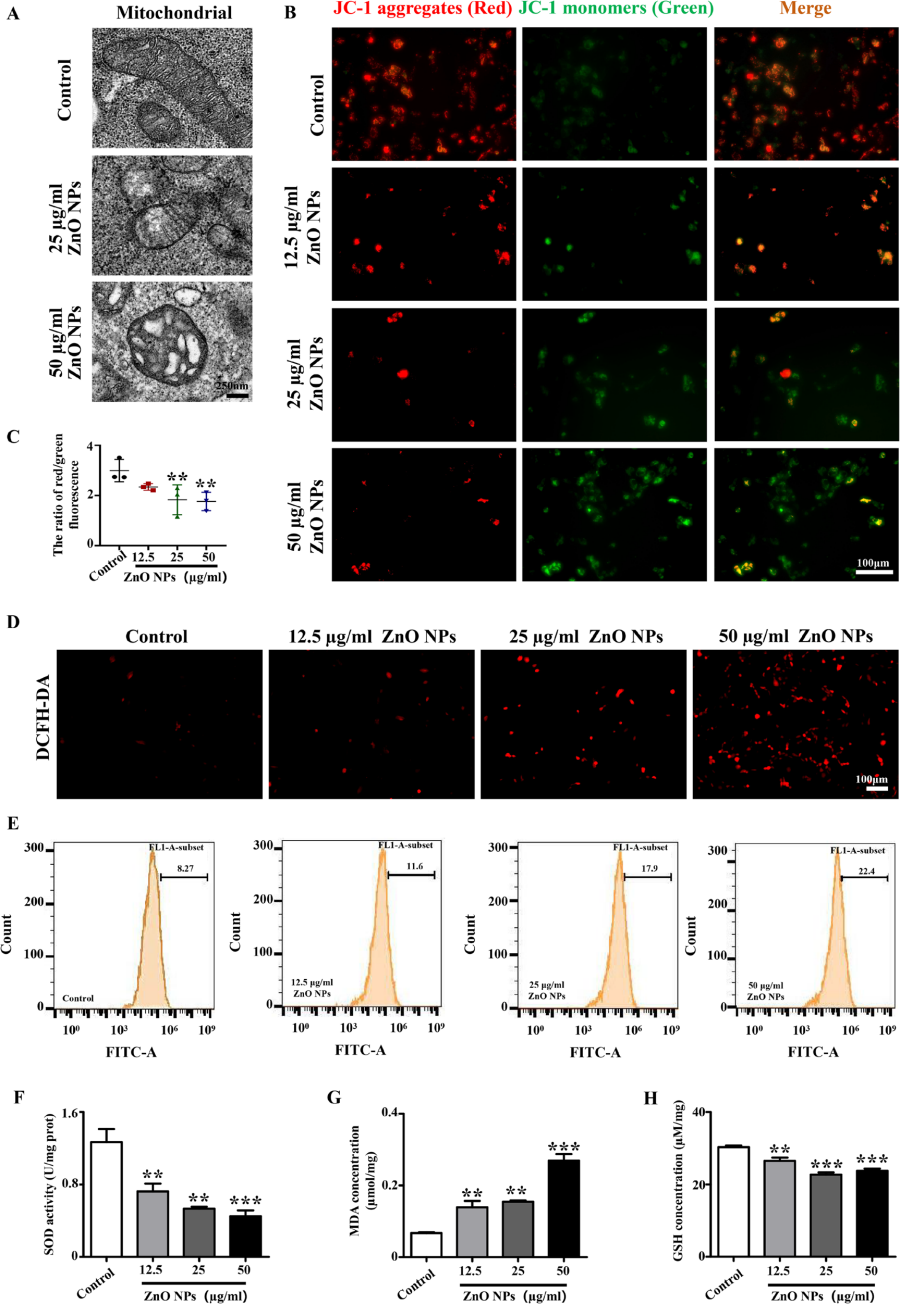
**Assessment of oxygen reactive species after ZnO NPs treatment** (**A**) TEM images showing the mitochondrial morphology of SH-SY5Y cells treaded with or without ZnO NPs for 6 h. (**B**) Representative images of JC-1 staining of SH-SY5Y cells treated with different concentrations of ZnO NPs for 6 h. (**C**) Bar chart showing the quantitative analysis of the ratio of red/green fluorescent intensity. (**D**) Representative images of DCFH-DA staining of SH-SY5Y cells treated with different concentrations of ZnO NPs for 6 h. (**E**) Flow cytometry analysis of ROS in SH-SY5Y cells treated with ZnO NPs at different concentrations for 6 h. (**F–H**) Bar charts showing the SOD activity, MDA concentration and GSH concentration of SH-SY5Y cells treated with or without ZnO NPs for 6 h. *P < 0.05, **P < 0.01, ***P < 0.001

**Fig. 8:**
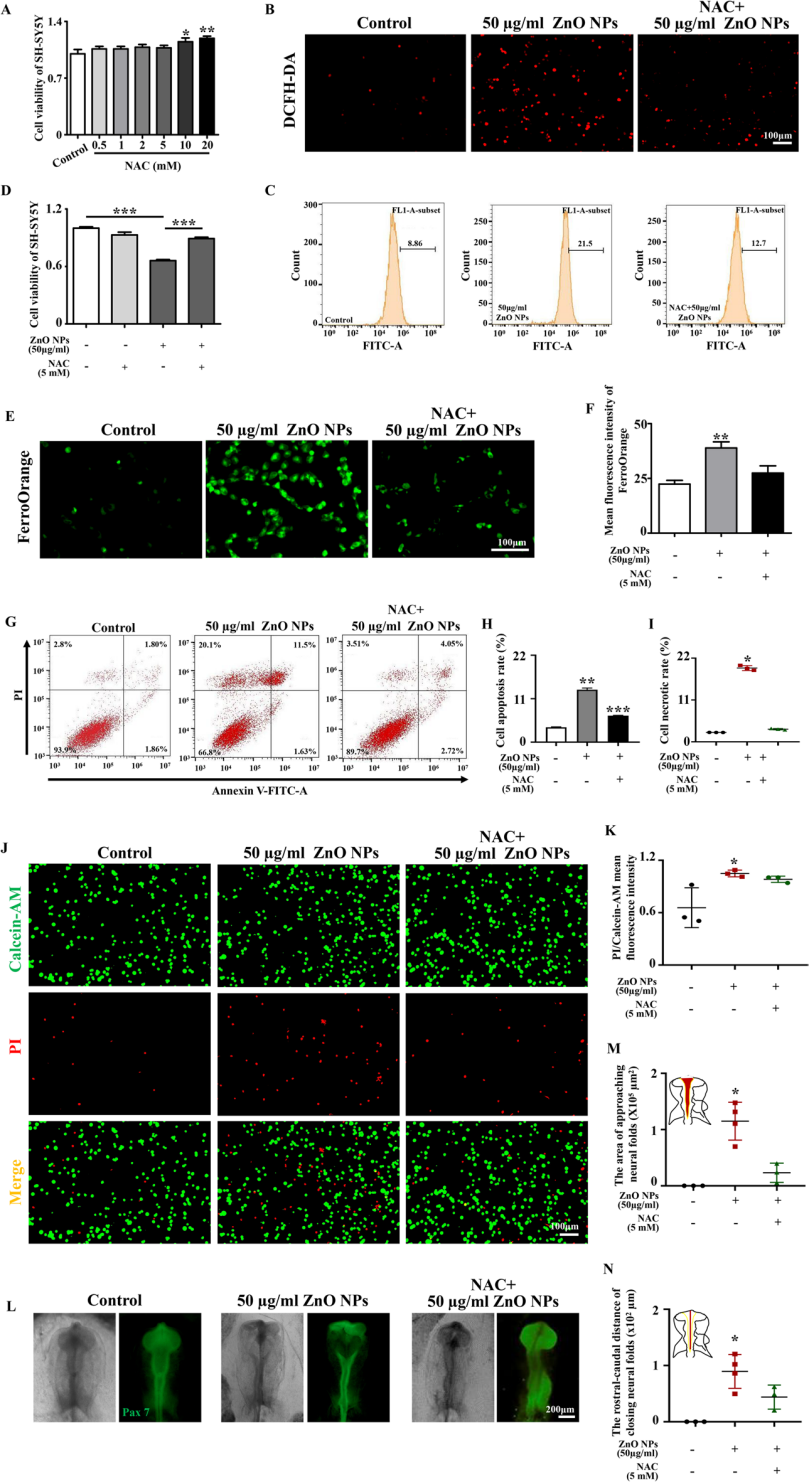
**Assessment of the rescue effect of NAC on ZnO NPs-induced cytotoxicity and neural tube defects** (**A**) Viability of SH-SY5Y cells after exposure to different concentrations of NAC for 6 h. (**B**) Representative images of DCFH-DA staining of SH-SY5Y cells after exposure to NAC or/and ZnO NPs for 6 h. (**C**) Flow cytometry analysis of ROS of SH-SY5Y cells after exposure to NAC or/and ZnO NPs for 6 h. (**D**) Viability of SH-SY5Y cells after exposure to NAC or/and ZnO NPs for 6 h. (**E**) Representative images of FerroOrange staining of SH-SY5Y cells after exposure to NAC or/and ZnO NPs for 6 h. (**F**) Bar chart showing the quantitative analysis of mean fluorescent intensity of FerroOrange staining. (**G**) Flow cytometry analysis of the apoptosis of SH-SY5Y cells after exposure to NAC or/and ZnO NPs for 6 h. (**H-I**) Bar charts showing the apoptosis rate or necrotic rate of SH-SY5Y cells after exposure to NAC or/and ZnO NPs for 6 h. (**J**) Representative images of Calcein-AM/PI double staining of SH-SY5Y cells treated with NAC or/and ZnO NPs for 6 h. (**K**) Bar chart showing mean fluorescence intensity of Calcein-AM/PI of SH-SY5Y cells treated with NAC or/and ZnO NPs for 6 h. (**L**) Representative images of chick embryo neural tubes in the control, ZnO NPs-treated and NAC + ZnO NPs-treated groups. (**M–N**) Bar charts showing the area of approaching neural folds and the rostral-caudal distance of closing neural folds in each group. *P < 0.05, **P < 0.01, ***P < 0.001

Mitochondria are the energy-producing organelles of cells and serve as the central hub for regulating cell death and cell survival (Collier et al. 2023). Mitochondrial damage is the initiation of cell death and is also the main pathway by which various nanoparticles disrupt cellular homeostasis (Horie and Tabei 2021). First, we found that exposure to ZnO NPs caused mitochondrial morphology to swell and mitochondrial cristae to disappear (Fig. 7A). Subsequently, the effect of ZnO NPs on mitochondrial membrane potential was assessed by JC-1 staining to evaluate the function of mitochondrial. The results showed a significant reduction in mitochondrial membrane potential of cells after ZnO NPs treatment compared to normal cells (Fig. 7B-C). This result indicates that ZnO NPs exposure caused mitochondrial damage. ROS are the main product of mitochondrial damage and can cause oxidative stress, further leading to inflammatory reactions and cell death (Liu et al. 2020; Zhang et al. 2023). We speculated that oxidative stress caused by mitochondrial damage may be the initial effector of ZnO NPs-induced cytotoxicity and neurotoxicity. By performing GO enrichment analysis on the DEGs screened by RNA-seq, we found that the neurotoxicity caused by ZnO NPs may be related to biological processes such as MAPK cascade, positive regulation of cell death, regulation of lipid metabolism, and inflammatory response, which preliminarily confirmed that ZnO NPs may exert their effects by affecting oxidative stress (Supplementary Fig. 4). DCFH-DA staining and ROS detection based on flow cytometry data further confirmed that ZnO NPs indeed caused increased levels of cellular oxidative stress (Fig. 7D-H). Zn^2+^ was confirmed to be involved in this process (Supplementary Fig. 5). NAC was utilized to explore the critical function of ZnO NPs-induced oxidative stress in causing cytotoxicity and nervous system developmental defects. To exclude interference from the cytotoxic effect of NAC itself, we first used CCK8 detection to screen out the NAC concentration that has no effect on cell activity for subsequent experiments (Fig. 8A). Thereafter, we confirmed that NAC did effectively reduce the expression of cellular oxidative stress levels (Fig. 8B-C). Further experiment revealed that NAC treatment could partially reverse the decrease in cell viability, cell ferroptosis and apoptosis induced by ZnO NPs (Fig. 8D-K). Moreover, NAC rescued the incomplete neural tube closure caused by ZnO NPs, which directly proved that oxidative stress is the major factor in the cytotoxicity and neurotoxicity induced by ZnO NPs (Fig. 8L-N).

## Discussion

Widespread use of ZnO NPs increases the risk of exposure to pregnant women. However, the understanding of the embryotoxicity and cytotoxicity of ZnO NPs and their interaction with the biological mechanisms remains limited. It is of significant necessity to conduct in-depth research on the embryotoxicity of ZnO NPs. Firstly, the results revealed that the ZnO NPs used here have small particle size, spherical/rod-like morphology, present a negative surface charge and display a certain degree of aggregation in different culture media. Studies have confirmed that small-sized nanoparticles exhibit higher toxic effects because they have a larger specific surface area and are easier to be taken up by cells (Xia et al. 2019). Negatively charged nanoparticles can induce cytotoxic effects by mediating the adsorption of cations (Ca^2+^) onto albumin (Chang et al. 2012). In addition, although the solubility of ZnO NPs in serum and EC culture is slightly higher than that in medium, this may be related to the rich proteins and small molecule complexing agents in serum and egg white (Bhalani et al. 2022). Regardless of the medium, the solubility of ZNO NPs did not exceed 15%, indicating that the solubility of ZnO NPs was limited and some of them still existed in the form of particles (Fig. 1).

The first thing to consider when studying the embryotoxic of ZnO NPs is their ability to penetrate cross placental barrier. We detected higher concentrations of Zn^2+^ in the placentas and embryos of pregnant mice injected with ZnO NPs through the tail vein, suggesting that ZnO NPs and the Zn^2+^ they release are not restricted by the placental barrier here. Chicken embryos are highly homologous to the human genome, and have the characteristics of short life cycle, transparent embryos, small size, and high sensitivity to poisons. We confirmed that ZnO NPs-treated chicken embryos showed restricted growth and an increased abnormal rate, indicating that ZnO NPs have embryotoxic effects (Fig. 2). It is worth noting that although embryonic mortality occurred after ZnO NPs treatment, there was no statistically significant difference in mortality rate between ZnO NPs treatment group and control group, which indicated that the negative impact of ZnO NPs exposure on embryonic development mainly manifests as teratogenicity rather than lethality under the experimental conditions of this study. The above result is consistent with the findings of previous studies, which have confirmed the strong association between environmental exposure to nanoparticles during pregnancy and adverse pregnancy outcomes, including low birth weight and preterm birth (Dadvand et al. 2013; Pedersen et al. 2013), risks of adverse pregnancy (Gómez-Roig et al. 2021; Yu et al. 2022), cardiovascular disease (Kim et al. 2020; Michel et al. 2023), respiratory problems (Bharadwaj et al. 2016; Hehua et al. 2017) and neurodevelopmental changes (Lertxundi et al. 2019; Song et al. 2021; Sunyer and Dadvand 2019). In addition, studies have confirmed that compared with the peri-implantation period of pregnancy, maternal mice in the organogenesis period are more susceptible to the effects of ZnO NPs, potentially leading to embryotoxicity (Teng et al. 2020). Therefore, it is necessary to conduct in-depth investigation of the specific target organ and mechanisms of early embryonic toxicity of ZnO NPs. The nervous system is highly sensitive to external stimuli during embryonic development. We used precise quantitative analysis to confirm that ZnO NPs cause closure defects in the early embryonic nervous system (Fig. 3). Considerable attention has been directed towards investigating the toxic effects of nanoparticles on neurogenesis, yet research specifically focusing on their impacts during early neurogenesis remains limited. Moreover, a comprehensive exploration of the underlying mechanisms is warranted. The data presented herein suggested that the ZnO NPs utilized in this study may possess potential toxic effects.

The regulation of cell death plays a crucial role in ensuring proper morphogenesis and cell number balance during central nervous system development. Excessive death of nerve cells may lead to a deficiency in cells critical for neural tube morphogenesis, resulting in neural tube closure defects (Moon and Xiong 2022; Pyrgaki et al. 2020). To further explore the potential mechanism underlying ZnO NPs-induced failure of embryonic neural tube closure, we first used SH-SY5Y cells to test the cell viability following treatment with ZnO NPs. This indicator serves as direct observation of cell fate and we aimed to use this experiment to comprehensively ascertain whether nerve cells could survive or die when exposed to ZnO NPs. Our results revealed a significant decrease in cell viability after ZnO NPs treatment, indicating a strong likelihood that extensive cell death may be the main reason for incomplete neural tube closure caused by ZnO NPs (Fig. 4). Nanoparticles can regulate the occurrence of various programmed cell death events depending on their dosage as well as physical and chemical properties (Mohammadinejad et al. 2019). We have shown in previous studies that ZnO NPs cause neuronal cell apoptosis by activating endoplasmic reticulum stress levels (Yan et al. 2021). However, apoptosis is only one of various programmed cell death modalities, and there are cross-regulation and cross-talk between apoptosis and other types of programmed cell death (Wang et al. 2023). In this study, we further confirmed that multiple programmed cell deaths are significant contributors in the neurotoxic effects of ZnO NPs, including apoptosis (Fig. 4), autophagy (Supplementary Fig. 3), and ferroptosis (Fig. 5). The above results are basically consistent with previous studies. Kim et al. reported that ZnO NPs induced necrosis and apoptosis of nerve cell by increasing LOX-mediated ROS levels (Kim et al. 2015). Roy et al. showed that ZnO NPs activated autophagy by inhibiting MTOR and inducing the phosphorylation of autophagy-related proteins and Bcl2 family proteins (Roy et al. 2014). Moreover, ZnO NPs induced ferroptosis in neural cells by selectively activating the JNK signaling pathway (Qin et al. 2020). The differential expression patterns of ferroptosis-related genes across various developmental stages and brain regions, suggest a role for ferroptosis and its associated genes in the development, maintenance of function, and aging processes within the nervous system (Zhou et al. 2022). The above research results provided more robust evidence supporting the involvement of various programmed cell death in incomplete neural tube closure caused by ZnO NPs. It is noteworthy that ZnO NPs were partially dissolved free Zn^2+^ after entering the body and cells. We have used Zn^2+^ chelators to confirm that Zn^2+^ play a role in the decline of cell activity caused by ZnO NPs (Yan et al. 2021). Here, we further verified whether Zn^2+^ are involved in oxidative stress and multiple modes of cell death caused by ZnO NPs. Our results revealed that the phenotypes induced by Zn^2+^ and ZnO NPs in cells are similar, suggesting that the free Zn^2+^ dissolved by ZnO NPs also contributes to the cytotoxicity induced by ZnO NPs. To sum up, the toxicity effect caused by ZnO NPs is the result of the combined effect of the particles themselves and the Zn^2+^ released from the nanoparticles (Fig. 6).

Based on our findings, we further raised the following research question: whether there exists a specific upstream pathway regulating the various programmed cell deaths induced by ZnO NPs. Our study demonstrates that ZnO NPs treatment lead to abnormalities in mitochondrial function and morphology Given the well-established association between mitochondrial dysfunction, oxidative stress and ROS (Park et al. 2021) and GO enrichment analysis data, we hypothesized that oxidative stress may be a key target for exploring different types of cell death induced by ZnO NPs (Supplementary Fig. 4). We found that ZnO NPs promoted the production of ROS, reduced mitochondrial membrane potential, and damaged the normal morphology and function of mitochondria, indicating that there is a causal relationship between ZnO NPs and oxidative stress (Fig. 7). Additionally, the involvement of Zn^2+^ in this process cannot be ignored (Supplementary Fig. 5). This finding is consistent with previous results that ZnO NPs induced a large amount of ROS production, leading to epigenotoxicity (Choudhury et al. 2017). Furthermore, co-treatment with the antioxidant NAC alongside ZnO NPs mitigated cytotoxicity and teratogenic effects on nervous system, highlighting oxidative stress serves as the primary trigger for ZnO NPs’ toxic effects (Fig. 8).

## Conclusions

This study used a mouse model to confirm that ZnO NPs can penetrate the placental barrier in the form of particles and ions during early pregnancy, and further used a chicken embryo model and in vitro cell experiments to confirm that ZnO NPs cause incomplete neural tube closure and cytotoxicity. Further research showed that oxidative stress leads to mitochondrial damage and excessive ROS production, triggering a variety of programmed cell deaths, resulting in an insufficient number of cells participating in the correct morphogenesis of neural tube and ultimately causing the incomplete closure of neural tube. In summary, we aimed to explore the impact of ZnO NPs on early embryos, a crucial yet understudied aspect. Our expectation is to arouse the awareness of pregnant women about exposure to ZnO NPs and provide important reference for the formulation of preventive measures for pregnant women exposed to ZnO NPs. While our findings provided valuable insights into the potential impact of ZnO NPs on early embryogenesis, it is acknowledged that extrapolating the results from animal models to human exposure remains a complex and nuanced task. Undoubtedly, it is necessary to further investigate the behavioral mechanisms of nanoparticles in the placenta in future studies, including the uptake, accumulation, and translocation processes of particles, to gain a more comprehensive understanding of their biological impacts and reveal the effects of ZnO NPs on human health.

## Supplementary Information

Below is the link to the electronic supplementary material.

Supplementary file1 (DOCX 2755 KB)

## Data Availability

The RNA-seq data used to support the findings of this study have been deposited in GEO.
