# Supplementary material for: Zinc oxide nanoparticles induces cell death and consequently leading to incomplete neural tube closure through oxidative stress during embryogenesis
Source: Cell Biol Toxicol. 2024 Jul 3;40(1):51. doi: 10.1007/s10565-024-09894-1 (PMC11222284; doi:10.1007/s10565-024-09894-1)
Supplement: Supplementary file 1 — Supplementary file1 (DOCX 2755 KB) [file 10565_2024_9894_MOESM1_ESM.docx]

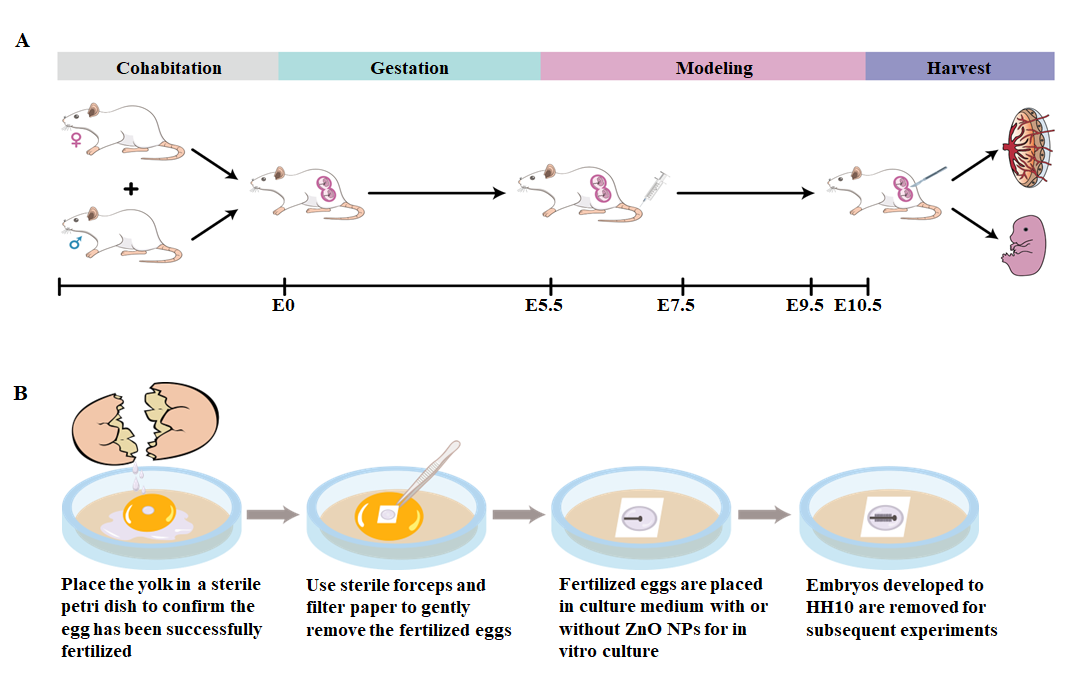


**Supplementary Figure 1 Schematic diagram of animal model modeling methods** (A) Schematic diagram showing the modeling method of pregnant mice exposed to ZnO NPs. (B) Schematic diagram of in vitro cultured chick embryos exposed to ZnO NPs.


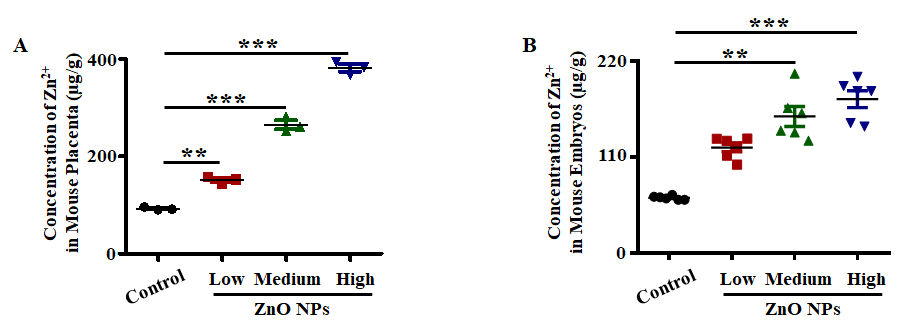


**Supplementary Figure 2 Assessment of Zn^2+^ concentration in placenta and embryos of pregnant mice after different concentrations of ZnO NPs treatment** (A) Scatter plot showing the concentration of Zn^2+^ in the placenta of the control and ZnO NPs-treated groups. (B) Scatter plot showing the concentration of Zn^2+^ in embryos in the control and ZnO NPs-treated groups. *P＜0.05, **P＜0.01, ***P＜0.001.


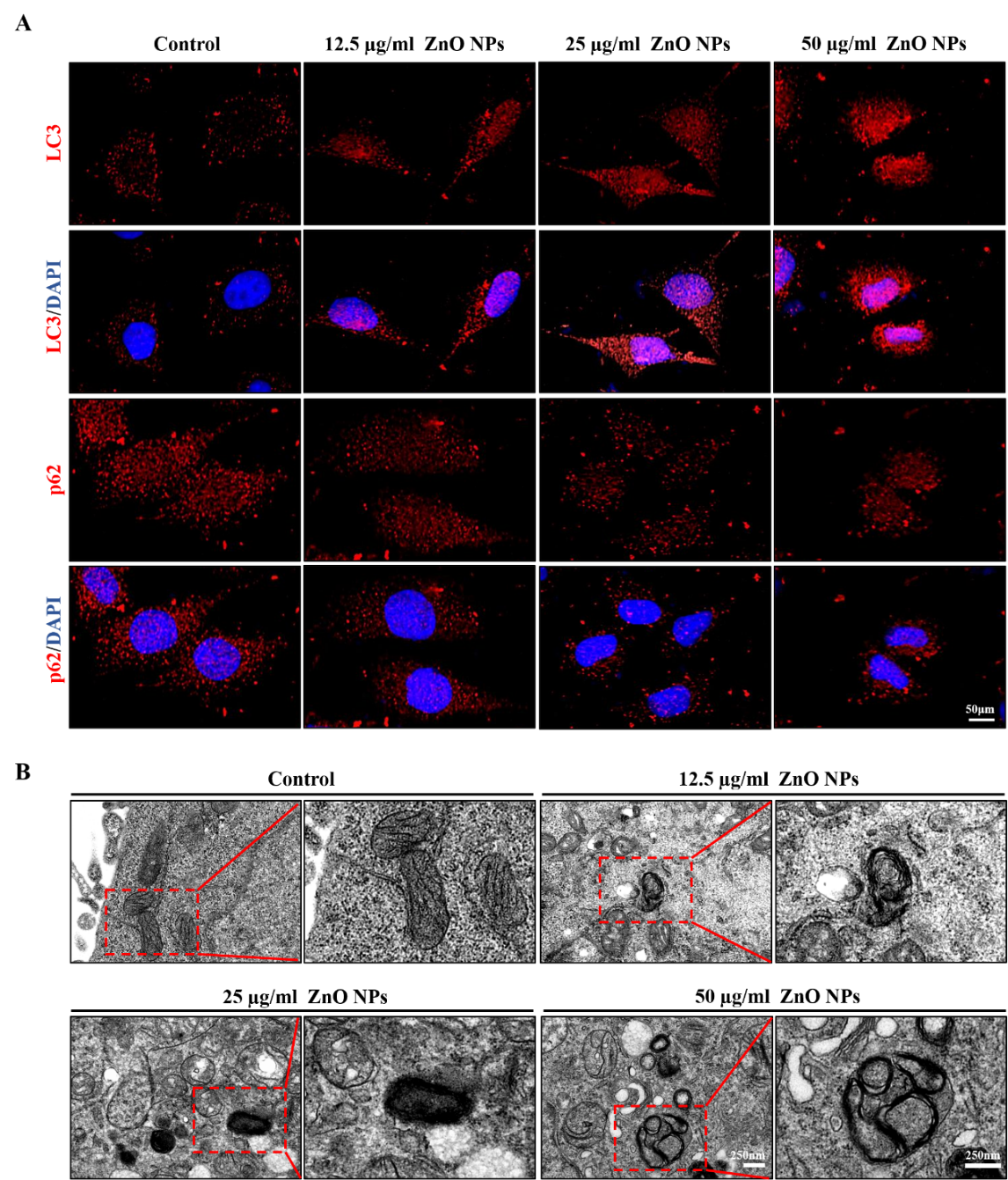


**Supplementary Figure 3 Assessment of autophagy activation of SH-SY5Y cells after different concentrations of ZnO NPs treatment** (A) Representative images of LC3 or p62 immunofluorescence staining of SH-SY5Y cells treated with different concentrations of ZnO NPs for 6 hours. (B) The typical TEM images of autophagosomes of SH-SY5Y cells treated with different concentrations of ZnO NPs for 6 hours. The High magnification images showed the autophagosomes with bilayer membrane structure.


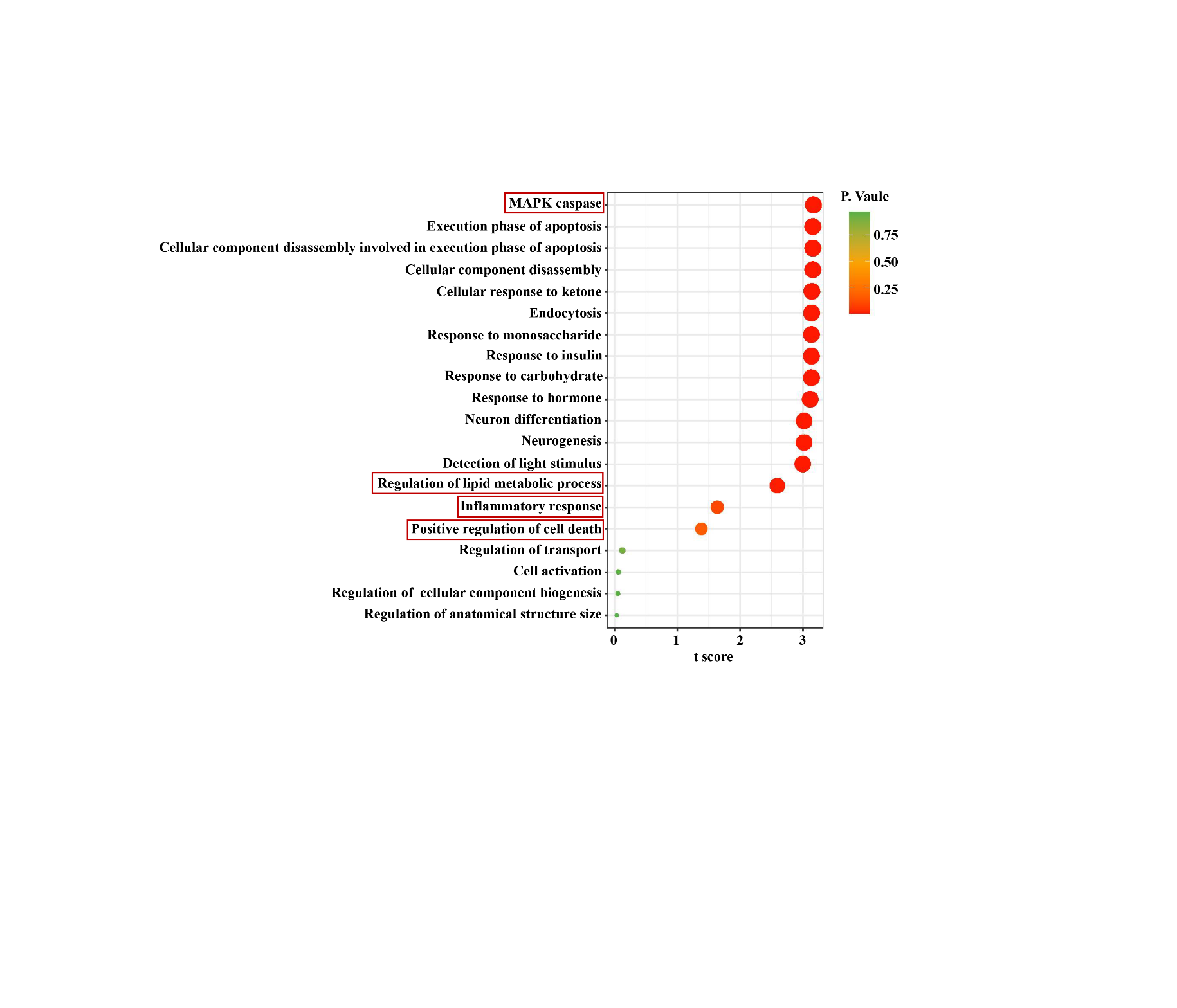


**Supplementary Figure 4 GO enrichment analysis of DEGs between control and ZnO NPs-treated chicken embryos.**


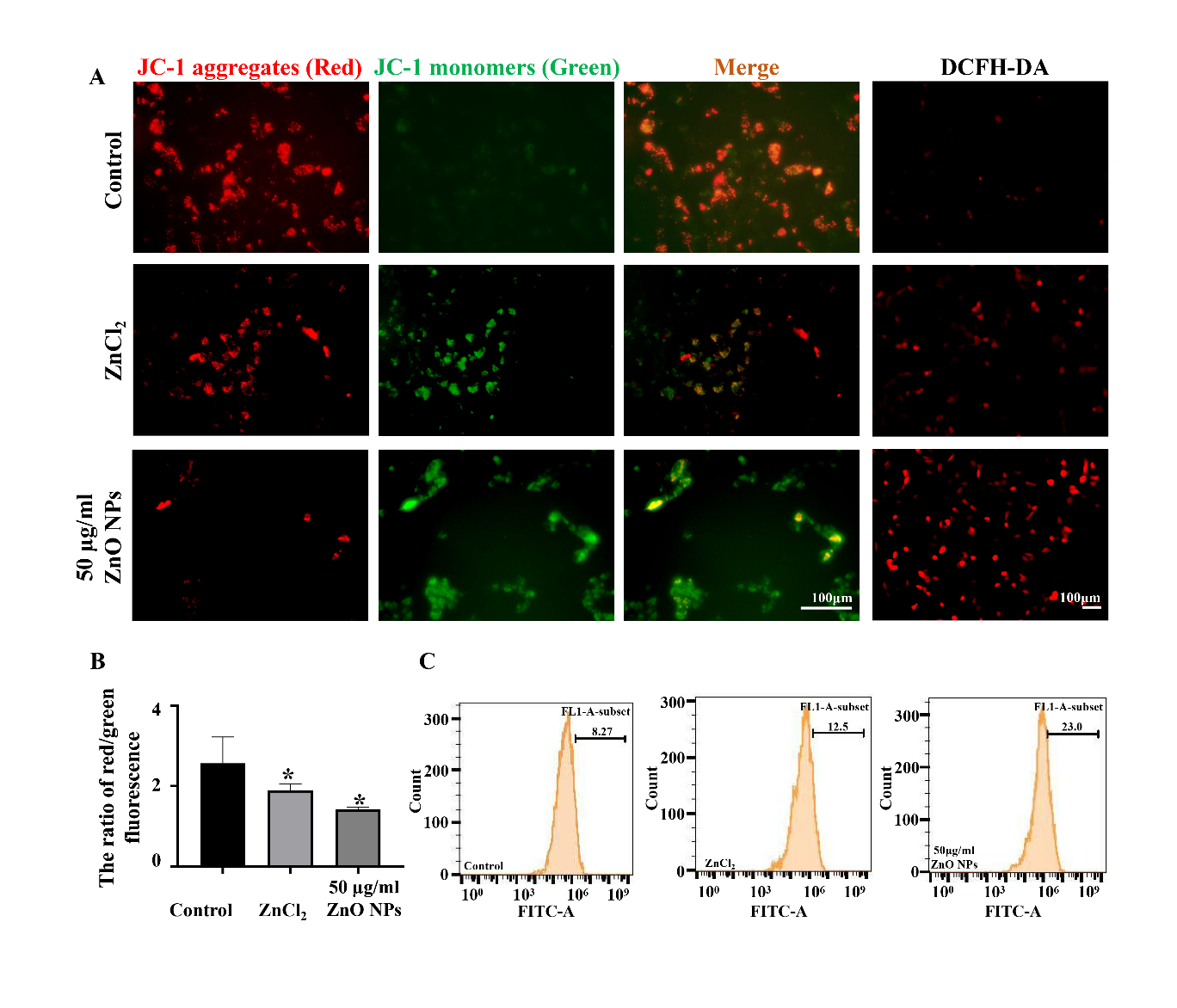


**Supplementary Figure 5 Assessment of oxygen reactive species after Zn^2+^ treatment** (A) Representative images of JC-1 staining of SH-SY5Y cells treated with Zn^2+^ or ZnO NPs for 6 hours. (B) Bar chart showing the quantitative analysis of the ratio of red/green fluorescent intensity. (C) Flow cytometry analysis of ROS in SH-SY5Y cells treated with Zn^2+^ or ZnO NPs for 6 hours. *P＜0.05, **P＜0.01, ***P＜0.001.
